# End-to-End Multimodal Sensor Dataset Collection Framework for Autonomous Vehicles

**DOI:** 10.3390/s23156783

**Published:** 2023-07-29

**Authors:** Junyi Gu, Artjom Lind, Tek Raj Chhetri, Mauro Bellone, Raivo Sell

**Affiliations:** 1 Department of Mechanical and Industrial Engineering, Tallinn University of Technology Tallinn, 12616 Tallinn, Estonia; raivo.sell@taltech.ee; 2 Intelligent Transportation Systems Lab, Institute of Computer Science, University of Tartu, 51009 Tartu, Estonia; artjom.lind@ut.ee; 3 Semantic Technology Institute (STI) Innsbruck, Department of Computer Science, Universität Innsbruck, 6020 Innsbruck, Austria; Tek-Raj.Chhetri@uibk.ac.at; 4 Center for Artificial Intelligence (AI) Research Nepal, Sundarharaincha 56604, Nepal; 5 FinEst Centre for Smart Cities, Tallinn University of Technology, 19086 Tallinn, Estonia; mauro.bellone@taltech.ee

**Keywords:** multimodal sensors, autonomous driving, dataset collection framework, sensor calibration and synchronization, sensor fusion

## Abstract

Autonomous driving vehicles rely on sensors for the robust perception of their surroundings. Such vehicles are equipped with multiple perceptive sensors with a high level of redundancy to ensure safety and reliability in any driving condition. However, multi-sensor, such as camera, LiDAR, and radar systems raise requirements related to sensor calibration and synchronization, which are the fundamental blocks of any autonomous system. On the other hand, sensor fusion and integration have become important aspects of autonomous driving research and directly determine the efficiency and accuracy of advanced functions such as object detection and path planning. Classical model-based estimation and data-driven models are two mainstream approaches to achieving such integration. Most recent research is shifting to the latter, showing high robustness in real-world applications but requiring large quantities of data to be collected, synchronized, and properly categorized. However, there are two major research gaps in existing works: (i) they lack fusion (and synchronization) of multi-sensors, camera, LiDAR and radar; and (ii) generic scalable, and user-friendly end-to-end implementation. To generalize the implementation of the multi-sensor perceptive system, we introduce an end-to-end generic sensor dataset collection framework that includes both hardware deploying solutions and sensor fusion algorithms. The framework prototype integrates a diverse set of sensors, such as camera, LiDAR, and radar. Furthermore, we present a universal toolbox to calibrate and synchronize three types of sensors based on their characteristics. The framework also includes the fusion algorithms, which utilize the merits of three sensors, namely, camera, LiDAR, and radar, and fuse their sensory information in a manner that is helpful for object detection and tracking research. The generality of this framework makes it applicable in any robotic or autonomous applications and suitable for quick and large-scale practical deployment.

## 1. Introduction

Nowadays, technological advancements such as deep learning and the introduction of autonomous vehicles (AVs) have altered every aspect of our lives and become an integral part of our economy. According to the Boston Consulting Group, the value of the AV industry in 2035 is projected to be $77 billion [1]. In addition, the Brookings Institution and IHS predict that by 2050, almost all users will possess AVs [2]. As AVs such as Tesla self-driving cars and AuVe Tech autonomous shuttles become more prevalent in our daily lives and an alternative to conventional vehicles, the safety and security concerns of AVs are growing [2]. Refining the current techniques can address concerns regarding AV safety and security. For instance, by enhancing object detection, we can enhance perception and reduce the probability of accidents.

Improving AV, particularly techniques such as object detection and path planning, requires field-collected AV data because field-collected AV data provide important insights, for example, human–machine interactive situations like merges, and unprotected turns [3], which are otherwise difficult to obtain from any simulated environment.

Moreover, diverse field-collected data can help AV technology to mature faster [4]. This is also why the amount of field-collected data for AVs is growing despite the availability of simulation tools such as CARLA [5] and SUMO (Simulation of Urban Mobility) [6]. Waymo’s open motion [3] and perception [7] dataset and the nuScenes [8] dataset are two examples.

However, collecting AV field data is a complex and time-consuming task. The difficulty stems from the multi-sensory (e.g., using multiple sensors such as camera, light detection and ranging (LiDAR), and radar) nature of AV environments, which are used to overcome the limitations of individual sensors. For example, the camera input can correct the abnormalities of inertial sensors [9]. However, the challenge lies in the fact that different sensors, such as LiDAR and radar sensors, have different sensing rates and resolutions and require the fusion of multimodal sensory data [10], thereby making the task of data collection even more difficult. For example, the LiDAR sensor can capture more than a million three-dimensional (3D) points per second, while the radar sensor has poor 3D resolution [11], which needs to be synchronized before use in other AV tasks such as object detection. Moreover, the data collection task is often performed alongside other regular duties, making it even more time-consuming and prone to error, which we conclude from our experience of iseAuto dataset collection [12].

With respect to the advantages of real-world field data, studies such as those by Jacob et al. [4] (see Section 2 for more) have focused on data collection frameworks for AVs. However, the work by Jacob et al. [4] does not consider the radar sensor; therefore, extra effort is required when the data are collected from a vehicle equipped with the radar sensor. Additional limitations of the work include the multi-sensor fusion of the camera, LiDAR, and radar data to provide rich contextual information. Muller et al. [13] leverage sensor fusion to provide rich contextual information like velocity, as in our work. However, the work of Muller et al. [13] does not include the radar sensor, and it is based on the CARLA simulator; hence, its effectiveness with real-world physical AVs is still being determined. Therefore, we present our work, an end-to-end general-purpose AV data collection framework featuring algorithms for sensor calibration, information fusion, and data space to collect hours of robot-related application that can generate data-driven models. The novelty of our dataset collection framework is that it covers the aspects from sensor hardware to the developed dataset that can be easily accessed and used for other autonomous-driving-related research. We provide detailed hardware specifications and the procedures to build the data acquisition and processing systems. Our dataset collection framework has backend data processing algorithms to fuse the camera, LiDAR, and radar sensing modalities together.

In summary, the contributions of this work are given below.

- We present a general purpose scalable end-to-end AV data collection framework for collecting high-quality multi-sensor radar, LiDAR, and camera data.
- The implementation and demonstration of the framework’s prototype, whose source code is available at: https://github.com/Claud1234/distributed_sensor_data_collector (accessed on 14 May 2023).
- The dataset collection framework contains backend data processing and multimodal sensor fusion algorithms.

The remainder of the paper is as follows. Section 2 reviews the autonomous driving dataset, the existing data collection frameworks, and the mainstream multimodal sensor systems related to autonomous data acquisition. Section 3 introduces the prior and post-processing of our dataset collection framework, including sensor calibration, synchronization, and fusion. Section 4 presents the prototype mount used for testing and demonstrating the dataset collection framework. Specifically, there are detailed descriptions of the hardware and software setups of the prototype mount and the architecture configurations of the system operating, data communication, and cloud storage. Section 5 evaluates the performance of our dataset collection framework based on the hardware of prototype we built for testing. Finally, Section 6 provides a summary and conclusion.

## 2. Related Work

Given the scope of this work, we present relevant studies distinguishing dataset collection frameworks for autonomous driving research from multimodal sensor systems for data acquisition. The reason is that many studies typically focus on one aspect or the other, while we intend to merge these concepts in a general-purpose framework.

### 2.1. Dataset Collection Framework for Autonomous Driving

Recently, data have been regarded as valuable property. For autonomous driving research, collecting enough data covering different weather and illumination conditions requires a lot of investment. Therefore, most research groups use open datasets for the experiments. For example, KITTI [14] has been one of the most successful open datasets for a long time. Because of the development of sensor technology and the increasing requirements for datasets to cover more weather and traffic conditions, the latest datasets, such as Waymo [7] and nuScenes [8], have adopted modern perceptive sensors and covered various scenarios. Other similar datasets include PandaSet [15], Pixset [16], and CADC [17]. Although public datasets offer researchers the convenience of obtaining data, their limitations in practical and engineering applications must be addressed. Most open datasets aim to provide well-synchronized, denoised, and ready-to-use data but are reckless in publishing the details of their hardware configurations and open sourcing the developing tools, which causes problems for other researchers to create the dataset they need. As a result, dataset collection frameworks are proposed. These frameworks focus on analyzing the feasibility of modern sensors and improving the system’s versatility on different platforms. Yan et al. [18] introduced a multi-sensor platform for vehicles to perceive their surroundings. Details of all the sensors, such as brand, model, and specifications, were listed in the paper. The robot operating system (ROS) was used for calibrating the sensors. Lakshminarayana et al. [19] focused on the protocols and standards for autonomous driving datasets. The author proposed an open-source framework to regularize datasets’ collection, evaluation, and maintenance, especially for their usage in deep learning. By contrast, the hardware cost was discussed in [4] as the budget is always critical for the large-scale deployment of a framework. Therefore, some researchers build the dataset pipelines by simulated vehicles and sensors to avoid the heavy investment of hardware purchase and repeated human–labor work, for example, manual object labeling. Moreover, simulation-based data generation frameworks can be used in applications that are difficult to demonstrate in the real world. For example, Beck et al. [20] developed a framework to generate camera, LiDAR, and radar data in the CARLA [21] simulator to reconstruct the autonomous-vehicles-involved accidents. Muller et al. [13] used the same CARLA platform to build a data collection framework to produce data with accurate object labels and contextual information. In summary, very few works provide a comprehensive end-to-end framework from hardware deployment to sensor calibration and synchronization, then to the backend camera–LiDAR–radar fusion that can be easily implemented into the end applications such as motion planning and object segmentation.

### 2.2. Multimodal Sensor System for Data Acquisition

The data acquisition of the modern autonomous and assisted driving system relies on the paradigm in which multiple sensors are equipped [22]. For autonomous vehicles, most of the onboard sensors serve the purposes of proprioception (i.e., inertia, positioning) and exteroception (i.e., distance measurement, light density). As our work concerns only the perceptive dataset collection, the review of multimodal data acquisition systems focuses on the exteroceptive sensors system for object detection and environment perception.

From the hardware perspective, exteroceptive sensors such as camera and LiDAR, and ultrasonic sensors, have to be installed in the exterior of the vehicles as they require a clear view field and less interference. For independent autonomous driving platforms, the typical solution is to install the sensors around the vehicles separately to avoid the body frame’s dramatic changes. The testing vehicle [23] has 15 sensors installed on the front, top, and rear sides to ensure the performance and appearance of the vehicles are not much affected. Other autonomous driving platforms with similar sensor layouts include [24,25]. Furthermore, shuttle-like autonomous vehicles such as Navya [26] and iseAuto [27] also adopt the same principle to fulfill the legal requirements for the real-traffic-deployed shuttle bus. In contrast, another sensor installation pattern integrates all perceptive sensors as an individual mount from the vehicle, which is often seen in the works related to dataset collection and experimental platform validation. The authors of [28,29] showcase the popular datasets in which all sensors are integrated. The experimental platforms examples that have detachable mounts onto the vehicles are given by the authors of [30,31].

The multimodal sensor systems’ software mainly involves the sensors’ calibration and fusion. Extrinsic and temporal calibration are two primary categories for multi-sensor systems. Extrinsic calibration concerns the transformation information between different sensor frames, and temporal calibration focuses on the synchronicity of multiple sensors operating at various frequencies and latencies. The literature on extrinsic calibration methodologies is rich. For example, An et al. [32] proposed a geometric calibration framework that combines the planar chessboard and auxiliary 2D calibration object to enhance the correspondences of 3D-2D transformation. Similarly, Domhof et al. [33] replaced the 2D auxiliary object with a metallic trihedral corner to provide strong radar reflection, which aims to reduce the calibration noise for radar sensors. In contrast to the calibration methods that employ specific targets, there are approaches dedicated to calibrating sensors without a target. Jeong et al. [34] utilized road markings to estimate sensor motions and then determined the extrinsic information of sensors. In [35], the authors trained a convolutional neural network to substitute humans to calibrate camera and radar sensors. The model automatically pairs radar point clouds with image features to estimate challenging rotational information between sensors. The studies of multimodal sensor fusion for autonomous driving perception and data acquisition were reviewed in [36,37]. Recent breakthroughs in deep learning have significantly inspired researchers to fuse the multimodal data streams in the level of feature and context [38,39]. On the other hand, neural-network-based fusion approaches require a significant amount of computing power. Remarkably, Pollach et al. [40] proposed fusing the camera and LiDAR data at a probabilistic low level; the simple mathematical computation consumes less power and causes low latency. The authors of [41] focused on the implementation feasibility of the multi-sensor fusion. Like our work, the authors developed a real-time hybrid fusion pipeline composed of a fully convolutional neural network and an extended Kalman filter to fuse the camera, LiDAR, and radar data. Cost efficiency is the crucial point in [42]; the study resulted in a method that relies on Microsoft Kinect to produce color images and 3D point clouds. However, this data acquisition and fusion system mainly works for road surface monitoring.

## 3. Methodology

**Figure 1:**
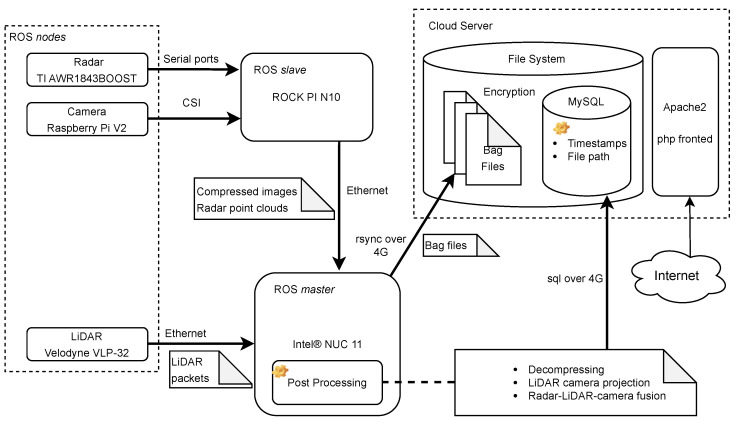
Overview of the framework architecture and data flow. The bold arrow-pointers denote data flow directions and corresponding protocols. Detailed descriptions of the sensor and other framework prototype hardware are in Section 4.

Our dataset collection framework primarily focuses on exteroceptive sensors mainly used in robotics for perception purposes, in contrast to sensors such as GPS and wheel-encoder that record the status information of the vehicle itself. Currently, one of the primary usages of the perceptive sensor data in the autonomous driving field is the obstacle-type-objects (cars, humans, and bicycles) [43] and traffic-type-objects (traffic signs and road surface) [44] detection and segmentation. The mainstream research in this field is fusing different sensory data to compensate sensors for each other limitations. There is already a large amount of research focusing on the fusion of camera and LiDAR sensors [45], but more attention should be given to the integration of radar data. Although LiDAR sensors outperform radar sensors from the perspective of point-cloud density and object texture, radar sensors have advantages in terms of moving object detection, speed estimation, and high reliability in harsh environments such as fog and dust. Therefore, this framework innovatively exploits the characteristics of radar sensors to highlight moving objects in LiDAR point clouds and calculate their relative velocity. The radar and LiDAR fusion result is then projected onto the camera image to achieve the final radar–LiDAR–camera fusion. Figure 1 presents the framework architecture and data flow overview. In summary, the framework is composed of three modules: sensors, processing units, and cloud server. The radar, LiDAR, and camera sensors used in the framework’s prototype are TI mmwave AWR1843BOOST, Velodyne VLP-32C, and Raspberry Pi V2, respectively. Sensor drivers are the ROS nodes and forward data to the connected computing unit. The main computer (ROS master) of the prototype is the Intel® NUC 11 with the Core™ i7-1165G7 Processor, and the supporting computer (ROS slave) is the ROCK PI N10. The ROS master and salve computers are physically connected by an Ethernet cable, and the ROS slave simply sends sensory data coming from the camera and the radar to the ROS master for post processing. The communication between the cloud server and the ROS master relies on the 4G network.

### 3.1. Sensor Calibration

For any autonomous vehicle’s perceptive system equipped with both passive (camera) and active (LiDAR, radar) sensors, referring to their capacity to measure the natural electromagnetic radiation of objects or the reflected energy emitted by the sensor. The sensor calibration is the calculation of the transformation matrices to bring all measurements in the same reference frame in order to associate different readings of the same objects coming from different sensors. A reliable calibration requires one to retrieve the intrinsic and extrinsic parameters.

#### 3.1.1. Intrinsic Calibration

**Figure 2:**
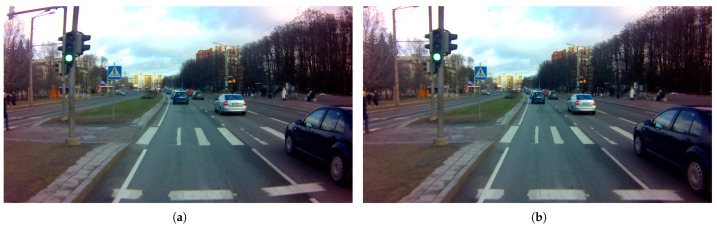
Comparing images obtained directly from the sensor to those that have been processed. (**a**) Raw distorted image obtained directly from the camera. (**b**) Rectified image.

The intrinsic calibration refers to the position and orientation of the sensor in real-world coordinates by which the relative coordinate for the features is detected by the sensor. Among all popular perceptive sensors in the autonomous driving field, there is already a significant amount of work related to the intrinsic calibration of the camera and LiDAR sensors [46,47]. LiDAR and camera are the primary sensors in this work to perceive the surrounding environment; therefore, they are the subject of intrinsic calibrations. Raspberry Pi V2 is a pinhole camera that is a well-known and widely used model [48,49]. The intrinsic calibration for the pinhole camera estimates the sensor’s internal parameters, such as focal length and distortion coefficients, that comprise the camera matrix. Referring to the classification in [50], we use the photogrammetric method to calibrate the Raspberry Pi V2 camera. This method relies on planner patterns with precise geometric information in the 3D real world. For example, using a checkerboard with known square dimensions, the interior vertex points of the squares are used during the calibration. In addition, a wide-angle lens (160°) was attached to the Raspberry Pi V2 camera, resulting in significant image distortion. Therefore, rectifying the images before implementing them into any post-processing is critical. The open-source ROS `camera_calibration’ package was used in this work to calibrate the camera sensor. The `camera_calibration’ package is built upon the OpenCV camera calibration and 3D reconstruction modules. It provides the graphic interface for parameter tuning and gives the results of the distortion coefficients, camera matrix, and projection matrix. Figure 2 compares the distorted image obtained directly from the camera sensor and the processed rectified image based on the camera’s intrinsic calibration results.

As a highly industrialized and intact-sealed product, Velodyne VLP-32C LiDAR sensors are usually factory calibrated before shipment. Referring to the Velodyne VLP-32C’s user manual, the range accuracy is claimed to be up to ±3 cm [51]. In addition, research works such as proposed by Glennie et al. [52] and Atanacio-Jiménez et al. [53] used photogrammetry or planar structures to further calibrate the LiDAR sensors to determine the error connection. However, considering the sparsity of the LiDAR points from spatial perspective, factory calibration of the Velodyne LiDAR sensors is sufficient for most of the autonomous driving scenarios. Therefore, no extra calibration work was conducted on the LiDAR sensors in our framework.

**Figure 3:**
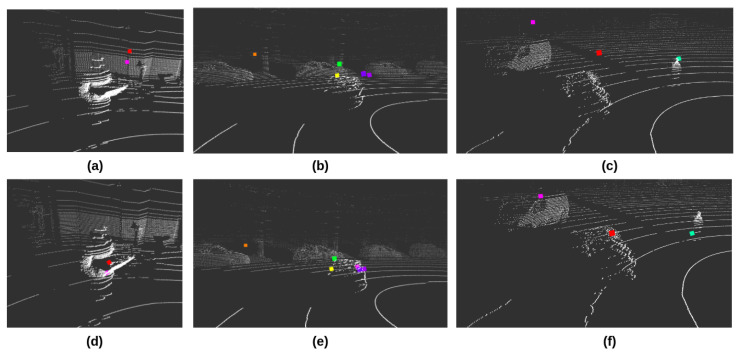
Performance of the LiDAR and radar extrinsic calibration in visualization. Color dots are radar points; white dots are LiDAR point clouds. The first row shows the relative locations of LiDAR and radar points without the extrinsic calibration. The second row is the results after applying the radar-LiDAR extrinsic calibration. The scene of (**a**,**d**) is indoor laboratory. (**b**,**e**) were captured in city’s urban area. (**c**,**f**) are in the open area outside the city.

Due to the characteristics of radar sensors in sampling frequency and spatial location, the calibration of radar sensors usually concentrates on the coordinate calibration to match the radar points and image objects [54]; points filtering to dismiss the noise and faulty detection results [55]; and error correction to compensate the mathematical errors in measurement [56]. The post-processing towards radar data in our work is overlaying radar points with the LiDAR point clouds. Therefore, the intrinsic calibration for radar sensors focuses on filtering out undesirable detection results and noise. A sophisticated method for noise and ineffective target filtering was proposed by [57], which developed intra-frame clustering and tracking algorithms to classify the valid objects signal from original radar data. The straightforward approach to calibrate the radar sensors is given in [55], which filtered the point clouds by the speed and angular velocity information; thus, the impact of stationary objects can be reduced in radar detection results. Our work implements a similar direct method to calibrate the TI mmwave AWR1843BOOST radar sensor. The parameters and thresholds related to the resolution, velocity, and Doppler shift were fine-tuned in the environments where autonomous vehicles are operated. Most of the points for static objects were filtered out in radar data (although the noise is inevitable in detection results). As a result, there is a reduction in the number of points representing the dynamic objects in each detection frame (shown in Figure 3). This issue could be addressed by locating and clustering the objects’ LiDAR points through the corresponding radar detection result. This part of the work will be detailed in Section 3.3.2.

#### 3.1.2. Extrinsic Calibration

For multimodal sensor systems, extrinsic calibration refers to the rigid transformation of the feature from one coordinate system to another, for example, the transformation of LiDAR points from the LiDAR coordinate frame to the camera coordinate frame. The extrinsic calibration estimates transformation parameters between the different sensor coordinates. The transformation parameters are represented as a 3 × 4 matrix containing the rotation (R) and translation (t) information. Extrinsic calibration is critical for sensor fusion post-processing in any multi-sensor system. One of the most important contributions of our work is the backend fusion of the camera, LiDAR, and radar sensors; thus, the extrinsic calibration was carried out between these three sensors. The principle of sensor fusion in our work is filtering out the moving objects’ LiDAR points by applying the radar points, augmenting the LiDAR point data with the object’s velocity readings from the radar, and then projecting the enhanced LiDAR point clouds data (that contain the location and speed information of the moving objects) onto camera images. Therefore, there is a need to extract the Euclidean transformation between the radar and LiDAR sensors and between the LiDAR and camera sensors. The standard solution is to extract the peculiar and sensitive features from the different sensors in the calibration environment. The targets used in extrinsic calibration usually have specific patterns such as planar, circular, and checkerboard for simplicity to match the features between point clouds and images.

Pairwise extrinsic calibration between the LiDAR and camera sensors in our work was inspired by the work [58]. The target for the calibration is a checkerboard with 9 and 7 squares in two directions. In practical calibration, several issues were raised and need to be noted:

- Before the extrinsic calibration, individual sensors were intrinsically calibrated and published the processed data as ROS messages. However, to have efficient and reliable data transmission and save bandwidth, ROS drivers for the LiDAR and camera sensors were programmed to publish only Velodyne packets and compressed images. Therefore, additional scripts and operations were required to handle the sensor data to match the ROS message types for the extrinsic calibration tools. Table 1 illustrates the message types of the sensors and other post-processing.
- The calibration relies on humans to match the LiDAR point and corresponding image pixel. Therefore, it is recommended to pick the noticeable features, such as the intersection of the black and white squares or the corner of the checkerboard.
- The point-pixel matches should be picked from the checkerboard in different locations covering all sensors’ full field of view (FOV). For camera sensors, ensure that the pixels from the image edges were selected. Depth varieties (the distance between the checkerboard and the sensor) are critical for LiDAR sensors.
- It is a matter of fact that human errors are inevitable when pairing points and pixels. Therefore, it is suggested to select as many pairs as possible and repeat the calibration to ensure high accuracy.

Compared with the abundant resource for pairwise LiDAR and camera extrinsic calibration, relatively little research addressed the multimodal extrinsic calibration that includes the radar sensors. Radar sensors usually have smaller FoV than the camera and LiDAR sensors, while they also lack elevation resolution and sparse point clouds. Therefore, poor informativeness is the primary challenge for radar’s extrinsic calibration. To address this problem, one of the latest references [59] proposed a two-step optimization method in which the radar data was reused in the second step to refine the extrinsic information gained from the first step calibration. However, the pursuit of our work is a universal pipeline that can be easily adapted to different autonomous platforms. Therefore, a toolbox bound with the standard ROS middleware is necessary to quickly deploy the pipeline system and execute the calibrations on autonomous vehicles. In our work, radar sensors were intrinsically calibrated to filter out most of the points for static objects. A minimum number of points were kept in each frame to represent the moving objects. An ROS-based tool was developed to compute the rotation and translation information between the LiDAR and radar coordinate frames. The calibration is based on the visualization of the Euclidean distance-based clusters of the point clouds data from two sensors. Corresponding parameters of the extrinsic calibration, such as Euler angles and displacement in X, Y, and Z directions, were manually tuned until the point cloud clusters overlapped. Please note that to properly calibrate the radar sensor, a specific calibration environment with minimal interference is required. Moreover, since the radar sensors are calibrated primarily to react to dynamic objects, the unique object in the calibration environment should move steadily and ensure the preferable reflective capability (TI mmwave radar sensors showed higher sensitivity to metallic surfaces than others during our practical tests). Figure 3 compares the result of LiDAR and radar extrinsic calibration in visualization. Each pair of figures in the column was captured from a specific environment related to the research and real-traffic deployment of our autonomous shuttles. The first column (Figure 3a,d) shows an indoor laboratory featuring a deficient interference; the only moving object is a human with a checkerboard. The second and third columns represent the outdoor environment where the shuttles were deployed. These two pairs also represent the different traffic scenarios. The second column (Figure 3b,e) is the city’s urban area, which has more vehicles and other objects (trees, street lamps, and traffic posts). The distance between the vehicles and sensors is relatively small; in this condition, radar sensors can produce more points. The third column (Figure 3c,f) is in the open area outside the city, which the vehicles run at a relatively high speed and far away from the sensors. The color dots represent the radar points, and the white dots are LiDAR point clouds data. The pictures in the first row illustrate the Euclidean distance between the LiDAR and radar point clouds before implementing the extrinsic calibration. The pictures in the second row show the results after the extrinsic calibration. By comparing the pictures in rows and columns, it is possible to see that the radar sensors produce less-noisy points data after the specific intrinsic calibration was implemented onto them. They are also more reactive to the metal surface and objects at a close distance. Moreover, after the extrinsic calibration of LiDAR and radar sensors, the alignment of the two types of sensors’ point clouds data was obviously improved, which is helpful for the further processing to identify and filter out the moving objects in LiDAR sensor’s point clouds data by the detection results of the radar sensor.

### 3.2. Sensor Synchronization

For autonomous vehicles that involve multi-sensor systems and sensor fusion applications, it is critical to address the synchronization of multiple sensors with different acquisition rates. The perceptive sensors’ operating frequencies are usually limited by their own characteristics. For example, as the solid-state sensor, cameras operate at high frequencies; on the contrary, LiDAR sensors usually scan at a rate of no more than 20 Hz because of the internal rotating mechanisms. Although it is possible to set the sensors to work at the same frequencies from the hardware perspective, the latency of the sensor data streams is also a problem for matching the measurements.

**Figure 4:**
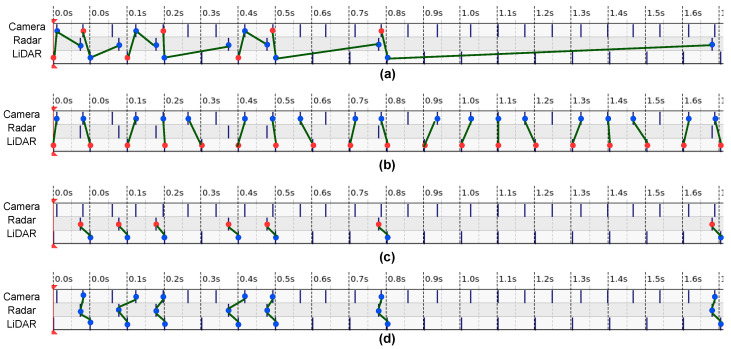
Illustrations of the sensor synchronization logic for message_filter and our algorithms. These illustrations are based on the real field data collected by our prototype. (**a**) shows the manner that message_filter carry out the multi-sensor synchronization, (**b**,**c**) show the individual LiDAR-camera and radar-LiDAR synchronization in our work, respectively. (**d**) is our final synchronized radar–LiDAR–camera message triplet. The messages of each sensor modality were represented by the blue points, the reference messages used for synchronization was highlighted in red. Green lines indicate the synchronized message sets. Please refer to Section 3.2 for more details.

In practical situations, it is not recommended to set all of the sensor frequencies identically. For example, reducing the frame rate of the camera sensors to match the frequencies of the LiDAR sensors means fewer images are produced. However, it is possible to optimize the hardware and communication setup to minimize the latency caused by the data transfer and pre-processing delays. The typical software solution to synchronize sensors matches the message headers’ closest timestamps at the end-processing unit. One of the most popular open-source approaches, ROS message_filter [60] developed an adaptive algorithm that first finds the latest message as a reference point among the heads of all topics (a term in ROS represents the information of sensing modality). The reference point was defined as the *pivot*; based on the *pivot* and a given time threshold, messages were selected out of all topics in the queues. The whole message-pairing process was shifted along the time domain. Therefore, the messages that cannot be paired (the difference of timestamps relative to other messages exceeds the threshold) would be discarded. One of the characteristics of this adaptive algorithm is that the selection of the reference message was not fixed into one sensor modality stream (shown in Figure 4a). For the systems with multiple data streams, the number of synchronized message sets are always reconciled to the frequency of the slowest sensor.

For any multi-sensor perceptive system, the sensor synchronization principle should correspond to the hardware configuration and post-processing of the sensor fusion. As discussed in Section 4.1.2 about the sensor configurations of our work, the camera sensor has the highest rate of 15 FPS, and the LiDAR sensor operates at 10 Hz. Both camera and LiDAR sensors work at a homogeneous rate, contrary to the heterogeneous radar sensors that only produce data when moving objects are in the detection zone. Therefore, as shown in Figure 4, depending on the practical scenarios, radar data can be sparser than the camera and LiDAR data and also can scatter unevenly along the time domain. In this case, the direct implementation of the synchronization algorithm [60] will cause significant data loss of the camera and LiDAR sensors. For the generic radar–LiDAR–camera sensor fusion in our work, we divide the whole process into three modules based on the frequencies of the sensors. The first module is the fusion of the LiDAR and camera data because these two sensors have constant rates. The second module is the fusion of the radar and LiDAR sensors as they both produce the point clouds data. Finally, the last module is the fusion of the result of the second module and the camera data, achieving the thorough fusion of all three sensory modalities.

To address the issues of the hardware setup and fulfill the requirement of fusion principles in our work, we develop a specific algorithm to synchronize the data of all sensors. Inspired by the work [60], our algorithm also relies on the timestamps to synchronize the messages. Instead of the *absolute timestamp* used in [60], we used the *relative timestamp* to synchronize the message sets. The definitions of two types of timestamps are:

- *Absolute timestamp* is the time when data were produced in sensors. It was usually created by the ROS drivers of the sensors and was written in the header of each message.
- *Relative timestamp* Relative timestamp represents the time data arrive at the central processing unit. It is the Intel® NUC 11 in our prototype.

Theoretically, the *absolute timestamp* should be the basis of the sensor synchronization as it represents the exact moment in which the data was created. However, *absolute timestamp* is not always applicable and has certain drawbacks in practical scenarios. First of all, it can be effectively implemented only if all sensors are capable of assigning the timestamp to each message on the fly, which is not always possible because of the computational capacity of the hardware, and software limitations. Regarding the cost consideration, some basic perceptive sensors are not integrated with the complex processing ability. For example, our prototype’s Raspberry Pi V2 camera has no additional computing unit to capture the timestamp. However, because it is a modular Raspberry camera sensor and is directly connected with the ROCK Pi computer through the CSI socket, the *absolute timestamp* is available in the header of each image message with the assistance of the ROCK Pi computer. On the other hand, the radar sensors used in the prototype have only serial communications with the computer, and there are no *absolute timestamps* for point clouds messages.

The second requirement for implementing the *absolute timestamp* is the clock synchronization between all of the computers in the data collection framework. There are two computers in our prototype; one serves as the primary computer performing all fundamental operations, and the second is the auxiliary computer used simply for launching the sensor and forwarding data messages to the primary computer. There is a need to synchronize the clock of all computers and sensor-embedded computing units to the precision of millisecond if using the *absolute timestamps* for sensor synchronization. An important aspect to be underlined in the specific field of autonomous driving is that sensor synchronization becomes even more important as the speed of the vehicle increases, causing distortion in sensors’ readings.

To simplify the deployment procedures of this data collection framework, our sensor synchronization algorithms trade off simplicity with accuracy by using the *relative timestamps*, which is the clock time of the primary computer when it receives the sensor data. Consequently, the algorithm is sensitive to the delay and bandwidth of the local area network (LAN). As mentioned in Section 4.1.1, all sensors and computers of the prototype are physically connected by internet cables and in the same Gigabyte LAN. In practical tests, before any payload was applied in the communication network, the average delay times between the primary computer and LiDAR sensor, as well as the secondary computer (camera and radar sensors), are 0.662 ms and 0.441 ms, respectively. By contrast, the corresponding delay times were 0.703 ms and 0.49 ms when data were transferred from the sensors to the primary computer. Therefore, the increasing time delay caused by transferring data in LAN is acceptable in practical scenarios. For example, the camera and LiDAR sensors’ time synchronization error of the Waymo dataset is mostly bounded from −6 to 8 ms [7].

The reference frame selection is another essential issue for sensor synchronization, especially for the acquisition systems with various types of sensors. The essential difference between message_filter and our algorithms is that the ROS-implemented message_filter selects the nearest upcoming message as a reference, while our algorithms fix the reference onto the same modality stream (compare the red dot locations in Figure 4a–c). Camera and LiDAR sensors have constant frame rates, but radar sensors produce data at a variable frequency, e.g., in the presence of a dynamic object. Therefore, in this case, the single reference frame is not applicable to synchronize all of the sensors. To address this problem, we divide the synchronization process in two steps. The first step is the synchronization of the LiDAR and camera data, as shown in Figure 4b. The LiDAR sensor was chosen as the reference; thus, the frequency of the LiDAR-camera synchronized message set is the same as the LiDAR sensor’s frame rate. The LiDAR-camera synchronization is continuous until the radar sensors capture the dynamic objects; in that case, the radar-LiDAR synchronization step begins, see Figure 4c. The radar sensor is the reference frame in the second synchronization step, which means that every radar message has a corresponding matched LiDAR message. As all LiDAR messages are also synchronized with the unique camera image, for every radar message, there is a thorough synchronized radar–LiDAR–camera message set (Figure 4d). The novelty of our synchronization method is separating the LiDAR and camera synchronization process from the whole procedure. As a result, we fully exploit the characteristics of density and consistency of the LiDAR and camera sensors while also keeping the possibility of synchronizing the sparse and variable information coming from radar sensors.

### 3.3. Sensor Fusion

Sensor fusion is critical for most autonomous-based systems as it integrates acquisition data from multiple sensors to reduce detection errors and uncertainties. Nowadays, most perceptive sensors have advantages in specific perspectives but also suffer drawbacks when working individually. For example, camera sensors may provide texture-dense information but are susceptible to changes in illumination; radar sensors can detect the reliable relative velocities of objects but struggle to produce dense point clouds; and state-of-the-art LiDAR sensors are supposed to address the limitations of camera and radar sensors but lack color and texture information. Relying on LiDAR data only makes object segmentation systems more challenging to carry out. Therefore, the common solution is combining the sensors to overcome the shortcomings of the independent sensor operation.

Camera, LiDAR, and radar sensors are considered the most popular perceptive sensors for autonomous vehicles. Presently, there are three mainstream fusion strategies: camera–LiDAR, camera–radar, and camera–LiDAR–radar. The fusion of camera and radar sensors has been widely utilized in industry. Car manufacturers combine cameras, radar, and ultrasonic sensors to perceive the vehicles’ surroundings. Camera–LiDAR fusion has often been used in deep learning in recent years. The reliable X-Y-Z coordinates of LiDAR data can be projected as three-channel images. The fusion of the coordinate-projected images and the camera’s RGB images can be carried out in different layers of the neural networks. Finally, the camera–LiDAR–radar fusion combines the characteristics of all three sensors to provide the excellent resolution of color and texture, precise 3D understanding of the environment, and velocity information.

In this work, we provide the radar–LiDAR–camera fusion as the backend of the dataset collection framework. Notably, we divide the whole fusion process into three steps. The first step is the fusion of the camera and LiDAR sensor because they work at constant frequencies. The second step is the fusion of the LiDAR and radar point clouds data. The last step combines the fusion result of the first two steps to achieve the complete fusion of the camera, LiDAR, and camera sensors. The advantages of our fusion approach are as follows:

- In the first step, camera–LiDAR fusion can have a maximum number of fusion results. Only a few messages were discarded during the sensor synchronization because the camera and LiDAR sensors have close and homogeneous frame rates. Therefore, the projection of the LiDAR point clouds to the camera images can be easily adapted to the input data of the neural networks.
- The second step fusion of the LiDAR and radar points grants the dataset the capability to filter out moving objects from dense LiDAR point clouds and be aware of objects’ relative velocity.
- The thorough camera–LiDAR–radar fusion is the combination of the first two fusion stage results, which consume little computing power and cause minor delays.

#### 3.3.1. LiDAR Camera Fusion

Camera sensors perceive the real world by projecting the objects onto the 2D image planes, while LiDAR point clouds data contain direct 3D geometric information. The study of [61] classified the fusion of 2D and 3D sensing modalities into three categories: high-level fusion, mid-level fusion, and low-level fusion. The high-level fusion first requires independent post-processing, such as object segmentation or tracking for each modality, then fuses the post-processing results; the low-level fusion is the integration of the basic information such as 2D/3D geometric coordinates and image pixel values in raw data, and the mid-level is an abstraction between high-level and low-level fusion, which is also known as feature-level fusion.

Our framework’s low-level backend LiDAR-camera fusion focuses on the spatial coordinate matching of two sensing modalities. Instead of deep learning sensor fusion techniques, we use traditional fusion algorithms for LiDAR-camera fusion, which means the input of the fusion process is the raw data, while the output is the enhanced data [62]. One of the standard solutions for low-level LiDAR-camera fusion is converting 3D point clouds to 2D occupancy grids within the FoV of the camera sensor. There are two steps of LiDAR-camera fusion in our dataset collection framework. The first step is transforming the LiDAR data to the camera coordinate system based on the sensors’ extrinsic calibration results; the process follows the equation:

(1) $$\left[\begin{array}{c} d_{x}\\ d_{y}\\ d_{z} \end{array}\right]=\left[\begin{array}{ccc} 1 & 0 & 0\\ 0 & cos(\theta_{x}) & sin(\theta_{x})\\ 0 & -sin(\theta_{x}) & cos(\theta_{x}) \end{array}\right]\left[\begin{array}{ccc} cos(\theta_{y}) & 0 & -sin(\theta_{y})\\ 0 & 1 & 0\\ sin(\theta_{y}) & 0 & cos(\theta_{y}) \end{array}\right]\left[\begin{array}{ccc} cos(\theta_{z}) & sin(\theta_{z}) & 0\\ -sin(\theta_{z}) & cos(\theta_{z}) & 0\\ 0 & 0 & 1 \end{array}\right]\left(\left[\begin{array}{c} a_{x}\\ a_{y}\\ a_{z} \end{array}\right]-\left[\begin{array}{c} c_{x}\\ c_{y}\\ c_{z} \end{array}\right]\right)$$

where $a_{x}$, $a_{y}$, and $a_{z}$ are the 3D point coordinates as seen from the original frame (before the transformation); $c_{x}$, $c_{y}$, and $c_{z}$ are the camera frame location coordinates; $\theta_{x}$, $\theta_{y}$, and $\theta_{z}$ are the Euler angles of the corresponding rotation of the camera frame; and $d_{x}$, $d_{y}$, and $d_{z}$ are the resulting 3D point coordinates as seen from camera frame (after transformation). The following step is the projection of the 3D points to 2D image pixels as seen from the camera frame; under assumption, the camera focal length and the image resolution are known, and the following equation performs the projection:

(2) $$\left[\begin{array}{c} u\\ v\\ 1 \end{array}\right]=\left[\begin{array}{ccc} f_{x} & 0 & \frac{W}{2}\\ 0 & f_{y} & \frac{H}{2}\\ 0 & 0 & 1 \end{array}\right]\left[\begin{array}{c} d_{x}\\ d_{y}\\ d_{z} \end{array}\right]$$

where $d_{x}$, $d_{y}$, and $d_{z}$ are the 3D point coordinates as seen from the camera frame; $f_{x}$ and $f_{y}$ are camera horizontal and vertical focal length (which is known from the camera specification or discovered during the camera calibration routine); $\frac{W}{2}$ and $\frac{H}{2}$ here are the coordinates of a principal point (the image center) derived from image resolution *W* and *H*; finally, *u* and *v* are the resulting 2D pixel coordinates. After transforming and projecting the 3D points into a 2D image, the filtering step removes all of the points that fall outside the camera view.

**Figure 5:**
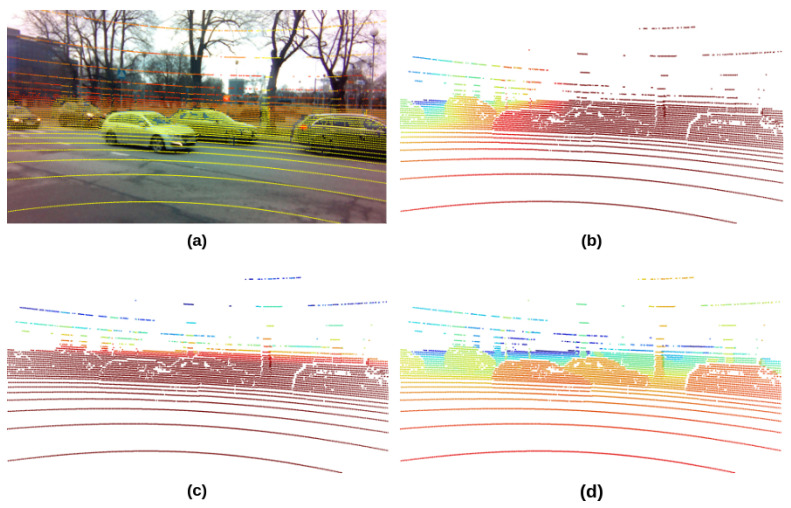
The projection of the LiDAR point clouds onto the camera plane in X, Y, and Z channels. (**a**) is RGB image, (**b**) is X channel projection, (**c**) is Y channel projection, and (**d**) is Z channel footprint. The color map of (**a**) is HSV, and (**a**–**c**) is JET.

The fusion results of each frame are saved as two files. The first is an RGB image with projected point clouds, as shown in Figure 5a. The 2D coordinate of LiDAR points was used to pick out the corresponding pixels in the image. The assignment of the pixel color is based on the depth information of the point, and the HSV colormap was used to colorize the image. The RGB image is the visualization of the projection result, which helps evaluate the alignment of the point clouds and image pixels. The second file contains the projected 2D coordinates and X, Y, and Z axis values of the LiDAR points within the camera view. All the information was dumped as a pickle file, which can be quickly loaded and adapted to other formats, such as array and tensor. The visual demonstrations of the information in the second file are shown in Figure 5b–d, which represents the LiDAR footprint projections in XY, YZ and XZ planes, respectively. The color of pixels in each plane is proportionally scaled based on the numerical 3D axes value of the corresponding LiDAR points.

The three LiDAR footprint projections are effectively formatted by, first, projecting the LiDAR points onto the camera plane and, second, assigning the value of the LiDAR axis to a projected point. The overall algorithm can be seen in the following subsequent steps:

1. LiDAR point clouds are stored in sparse triplet format $L^{3\times N}$, where *N* is the number of points in LiDAR data.
2. The transformation of LiDAR point clouds to the camera reference frame occurs through the multiplication of the LiDAR matrix L with the *LiDAR-to-camera* transformation matrix $T_{lc}$.
3. The transformed LiDAR points are projected to the camera plane, preserving the structure of the original triplet structure; in essence, the transformed LiDAR matrix $L_{T}$ is multiplied by the camera projection matrix $P_{c}$; as a result, the projected LiDAR matrix $L_{pc}$ now contains the LiDAR point coordinates on the camera plane (pixel coordinates).
4. The camera frame width *W* and height *H* are used to cut off all the LiDAR points that fall outside the camera view. In consideration of the projected LiDAR matrix $L_{pc}$ from the previous step, we calculate the matrix row indices where the values satisfy the following:
  - $0<=X_{pc}<W$
  - $0<=Y_{pc}<H$
  - $0<=Z_{pc}$
  The row indices where $L_{pc}$ satisfies the expressions are stored in an index array $L_{idx}$; the shapes of the $L_{T}$ and $L_{pc}$ are the same, therefore it is secure to apply the derived indices $L_{idx}$ to both the camera-frame-transformed LiDAR matrix $L_{T}$ and the camera-projected matrix $L_{pc}$.
5. The resulting footprint images XY, YZ, and XZ are initialized following the camera frame resolution W×H and subsequently populated with black pixels (zero value).
6. Zero-value footprint images are populated as follows:
  - $XY\left[L_{\mathrm{idx}}\right]=L\left[L_{\mathrm{idx}},0\right]$
  - $YZ\left[L_{\mathrm{idx}}\right]=L\left[L_{\mathrm{idx}},1\right]$
  - $XZ\left[L_{\mathrm{idx}}\right]=L\left[L_{\mathrm{idx}},2\right]$

The Algorithm 1 illustrates the procedures described above.
**Algorithm** **1** LiDAR transposition, projection populating the images 1:L[3×N]← nextFrame 2:$T_{\mathrm{lc}}\leftarrow$ conf 3:$P_{c}\leftarrow$ conf 4:$L_{\mathrm{pr}}=L*T_{\mathrm{lc}}*P_{c}$ 5:$L_{idx}=argwhere\left(L_{pr}>=\left\{0,0,0\right\}\;\&\;L_{pr}<\left\{W,H,+\infty \right\}\right)$ 6:XY[W×H]←0 7:YZ[W×H]←0 8:XZ[W×H]←0 9:$XY\left[L_{\mathrm{idx}}\right]=L\left[L_{\mathrm{idx}},0\right]$10:$YZ\left[L_{\mathrm{idx}}\right]=L\left[L_{\mathrm{idx}},1\right]$11:$XZ\left[L_{\mathrm{idx}}\right]=L\left[L_{\mathrm{idx}},2\right]$

#### 3.3.2. Radar LiDAR and Camera Fusion

This study uses millimeter wave (mmwave) radar sensors installed on the prototype mount. The motivations of equipping mmwave radar sensors on autonomous vehicles are to robustify perception against adverse weather; to prevent individual sensor failures; and, most importantly, to measure the target’s relative velocity based on the Doppler effect. Currently, mmwave radar and vision fusion can be seen as a promising approach to improve object detection [63]. However, most research relies on advanced image processing methods to extract the features from the data. Therefore, an extra process is needed to process the radar points into an image-like data format. Moreover, data conversion and deep-learning-based feature extraction consume a great amount of computing power and require noise-free sensing streams. As radar and LiDAR data are both represented as 3D Cartesian coordinates, the most common solution for data fusion is simply applying a Kalman Filter [64]. Another example work [65] first converted the 3D LiDAR point clouds to virtual 2D scans and then converted the 2D radar scans to 2D obstacle maps. However, their radar sensor is the mechanical pivoting radar, which differs from our mmwave radar sensors.

**Figure 6:**
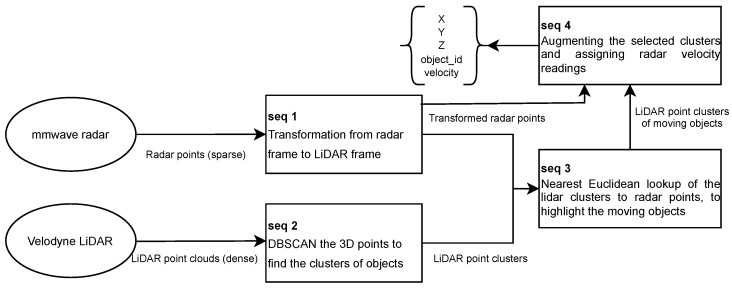
The workflow of radar-LiDAR fusion procedures.

In our work, the entire radar–LiDAR–camera fusion operation is divided into two steps. The first step is the fusion of radar and LiDAR sensors. The second step uses the algorithms proposed in Section 3.3.1 to fuse the first step’s results and camera images. As discussed in Section 3.1, we calibrate the radar sensors primarily reactive to the dynamic objects. As a result, the principle of the radar-LiDAR fusion in our work is selecting the LiDAR point clouds of the moving objects based on the radar detection results. Figure 6 illustrates four subsequent procedures of the radar-LiDAR fusion. The first involves transforming the radar points from the radar frame coordinate to the LiDAR frame coordinate. Corresponding transformation matrices are attained from the extrinsic sensor calibration. The second involves applying the density-based spatial clustering of applications with noise (DBSCAN) algorithm to the LiDAR point clouds to cluster out the points that potentially represent the objects [66]. The third involves looking up the nearest LiDAR point clusters for the radar points that were transformed into the LiDAR frame coordinate. The fourth involves marking out the selected LiDAR point clusters in raw data (arrays contain the X, Y, and Z coordinate values) and appending the radar’s velocity readings as an extra channel for selected LiDAR point clusters (or −∞ in case a LiDAR point belongs to no cluster).

**Figure 7:**
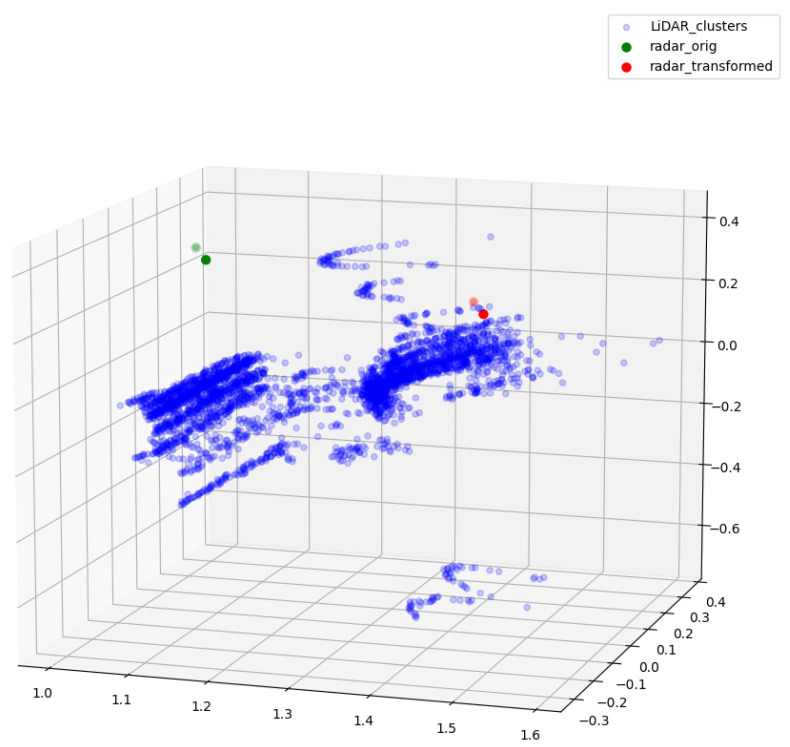
Relative locations of the original radar points (green), transformed radar points (red), and LiDAR point clusters of the moving object (blue). Scenario taken from a sequence similar to Figure 8.

Figure 7 demonstrates the relative locations of the original and coordinate-transformed radar points, and the results of the radar-LiDAR fusion in our work (LiDAR point clusters of the moving objects). The reference frame for the point-cloud scattering is the one positioned at the center of the LiDAR sensor. Green dots symbolize the original radar points, whereas red dots stand for the radar points transformed to the LiDAR frame coordinate, which are the result of the first subsequent of our radar-LiDAR fusion. Blue dots are the LiDAR point of the moving objects. The selection of the LiDAR point clusters, representing the detected moving object, relies on the nearest neighbor lookup based on the Euclidean distance metric that takes coordinate-transformed radar points as the reference. Due to inherent characteristics and post-intrinsic calibration, radar sensors in our prototype only produce a handful of points for moving objects in each frame, which means the computation of the whole radar-LiDAR fusion operation is computationally efficient and can be executed on the fly.

**Figure 8:**
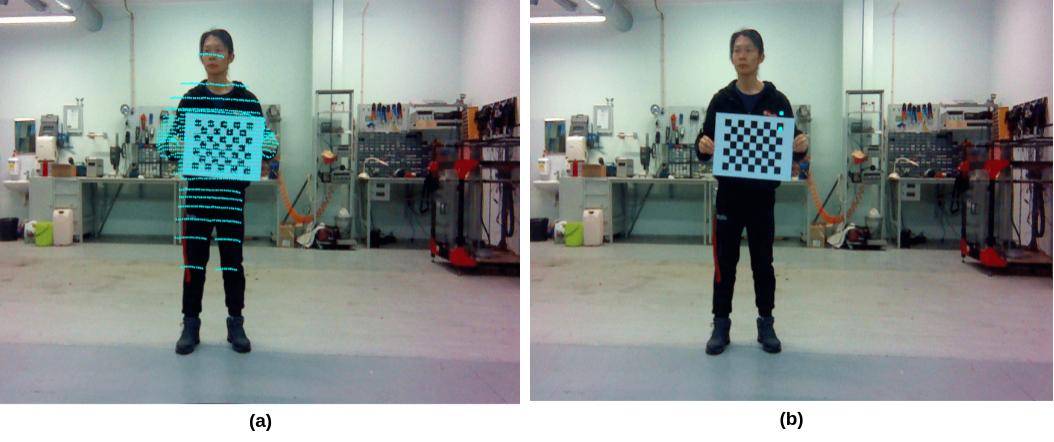
Illustration of the radar-LiDAR-camera. (**a**) Overposed LiDAR point cluster as extracted using the radar point as a reference, and (**b**) projection of the radar data onto the camera image.

The second step of the radar–LiDAR–camera fusion is the continuous process toward the results of the first step of radar-LiDAR fusion. The LiDAR point clusters that belong to the moving objects will be projected onto the camera plane. Figure 8a visualizes the final outcome of the radar–LiDAR–camera fusion in our dataset collection framework. LiDAR point clouds representing moving objects were filtered from the raw LiDAR data and projected onto the camera images. For each frame, moving objects’ LiDAR point clusters were dumped as a pickle file containing 3D-space and 2D-projection coordinates of the points and the relative velocity information. Because of the sparsity of the radar points data, the direct projection of the radar points onto camera images has very little practical significance (see Figure 8b). In fact, only two radar points are shown in this frame, and for this reason the significant result is the LiDAR point cluster in Figure 8a.

## 4. Prototype Setup

This section presents our prototype for demonstrating and testing the dataset collection framework. In addition, we provide detailed introductions of the hardware installation, framework operating system, data transferring protocols, and architecture of cloud services.

### 4.1. Hardware Configurations

**Figure 9:**
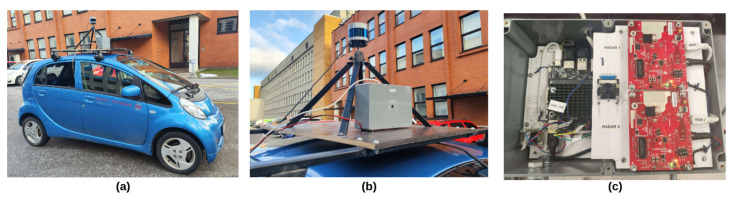
The prototype of the dataset collection framework. (**a**) is the Mitsubishi i-MiEV testing vehicle with the sensors mounted on the top. (**b**) shows the locations of sensors and other hardware. (**c**) shows the inside of the waterproof shell, which has one supporting computer, one camera, and two radar sensors.

This work aims to develop a general framework for autonomous vehicles to collect sensory data when performing regular duties. In addition, process the data in formats that can be used in other autonomous-driving-related technologies, such as sensor-fusion-based object detection and real-time environment mapping. A Mitsubishi i-MiEV car was equipped with a mount on the top (shown in Figure 9b), and all the sensors were attached to the mount. To increase the hardware compatibility, two processing units were used for the prototype mount to initiate the sensors and collect data. The main processing unit, which initiates the LiDAR sensor and handles the post-processing of the data, is located inside the car. Another supporting processing unit connected to the camera and radar sensors stays on the mount (outside the car and protected by water-dust-proof shells). The dataset collection framework was operated upon by the ROS; all sensory data were captured in corresponding ROS formats.

#### 4.1.1. Processing Unit Configurations

Three requirements have to be satisfied for the processing units and sensor components for the prototype:

- All the sensors must be modular, in a manner that they can work independently and can be easily interchanged. Therefore, there is a need for independent and modular processing units to initiate sensors and transfer the data.
- Some sensors have hardware limitations. For example, our radar sensors rely on serial ports for communication, and the cable’s length affects the communication performance in practical tests. A corresponding computer for radar sensors has to stay nearby.
- The main processing unit hardware must provide enough computation resources to support complex operations such as real-time data decompression and database writing.

The main computer for the prototype is an Intel® NUC 11 with a Core™ i7-1165G7 Processor, and the supporting computer is a ROCK PI N10 with four Cortex-A53 processors. The main computer is connected to the LiDAR sensor and 4G router, subscribes to data streams of the camera and radar sensors (published by supporting processing unit), carries out the data post-processing, and then sends corresponding information to the remote database server. The supporting computer is connected to the camera and radar sensors and stays inside the water-dust-proof shell that protects other electronic devices outside the vehicle (shown in Figure 9c). The communication between the two computers relies on the LAN.

#### 4.1.2. Sensor Installation

All the sensors installed in the prototype have been used and tested by other autonomous-driving-related projects [67,68] in the autonomous driving lab. Four perceptive sensors are installed on the prototype mount: one LiDAR, one camera, and two radars.

Currently, LiDAR and camera sensors are the mainstream in the autonomous driving field. Although it is a relatively new technology, LiDAR has become an essential sensor for many open datasets [28,69] and autonomous driving platforms [23,70]. The trend in the research community towards LiDAR sensors is using high-resolution models to produce the dense point clouds data; the maximum number of the vertical channels of the LiDAR sensors can be 128, and the range can reach 240 m. Correspondingly, dense point clouds data requires a large amount of bandwidth transference and processing power. To explicitly demonstrate our dataset collection framework and simplify the hardware implementation process, the LiDAR sensor used on the prototype is the Velodyne VLP-32C, which has 32 laser beams and vertically 40° FoV. The LiDAR sensor was connected to the main computer (NUC 11) by ethernet cable.

Camera sensors have a long developing history and are still important in modern autonomous driving technologies because of their advantages, such as reliability and cost-effectiveness. Moreover, the recent breakthrough of vision-based deep learning algorithms for object detection and segmentation has brought the researchers’ focus back to the camera sensor. Therefore, it is critical for our framework to have the capability to produce and process the camera data. Since the supporting computer (Rock Pi) has the specific camera serial interface (CSI) socket, the choice of the camera sensor for the prototype mount is the Raspberry Pi V2 camera with a wide angle (160° diagonal FoV). The camera can capture 3280 × 2464 pixel static images and up to 90 Hz video mode in resolution 640 × 480.

Radar sensors have been comprehensively used on commercial cars for driving assistance. However, most of the radar-based assistant functions, such as collision warning and distance control, simply use the character of reflectivity of the radar sensors. Another iconic characteristic of the mmwave radar sensors is their capability to detect moving objects. The velocity of the moving objects can be derived based on the Doppler effect. In addition, compared with the LiDAR sensors’ point clouds data that homogeneously project to all surrounding objects and whose total number of points are counted in millions, radar sensors can only focus on moving objects and produce much more sparse point clouds data that is friendly to the data transfer and storage. As mentioned in Section 3, one of the contributions of our work is using the mmwave radar sensors to detect moving objects and enhance them in LiDAR and camera data. The testing mmwave radar sensor used for our data collection framework is Texas Instruments mmwave AWR1843BOOST with 76 to 81 GHz frequency coverage and 4 GHz available bandwidth.

Figure 9 and Table 2 show all sensors’ aspects and detailed specifications. Please note that the parameters in Table 2 are the maximum values sensors can manage under the firmware and developing kit versions used in our experiments. In practical terms, the resolution and frame rate were reduced to meet the bandwidth and computation power limits. The LiDAR sensor operates at 10 Hz, and the camera runs at 15 Hz with a resolution of 1920 × 1080. Moreover, the maximum unambiguous range of the radar sensor was set as 30 m, and the maximum radial velocity is 15.37 m/s. The corresponding resolution of range and radial velocity is 0.586 and 0.25 m, respectively. To address the common problems of the radar sensors, such as sparse and heterogeneous point clouds, and a high level of uncertainty and noise for moving object detection, there are two radars installed next to each other in the box, as shown in Figure 9c. Camera and radar sensors are in close proximity, so the image and points data are consistent with each other and produce accurate perceptive results. Unlike the camera and radar sensors with limited horizontal FoV, LiDAR sensors have 360° horizontal views. To fully utilize this characteristic of the LiDAR sensors, one of the most popular methods is installing multiple camera and radar sensors in all directions. For example, the acquisition system of Apolloscape [29] has up to six video cameras around the vehicle; multiple LiDAR and radar sensors were installed in pairs in [23] to cover most of the blind spots. It is a fact that the prototype mount in this work only records camera and radar data in front view. However, the scope of this work is demonstrating a generic framework for data collection and enhancement. Future work will include setting more camera–radar modules in different directions.

### 4.2. Software System

The software infrastructure of the dataset collection framework was adapted from the iseAuto, the first autonomous shuttle deployed in real-traffic scenarios in Estonia. Based on the ROS and Autoware [71], the software infrastructure of the iseAuto shuttle is a generic solution for autonomous vehicles for sensor launching, behavior making, motion planning, and artificial intelligence-related tasks. The infrastructure contains a set of modules, including human interface, process management, data logging, and transferring. Like the iseAuto shuttle, the pipeline of the dataset collection framework was operated upon the ROS and captures all the sensory data in the corresponding ROS formats. As ROS is designed with distributed computing capability, multiple computers can run the same ROS system with only one master; thus, the ROS data from different slaves is visible to the whole network. In this work, the supporting computer connected to the camera and radar sensors works as an ROS slave, and the main computer hosts the ROS master. Complete and bi-directional connectivity exists between the main and supporting computers on all ports. In addition, the Gigabyte Ethernet connection guarantees low latency to transfer the camera and radar data from the supporting computer to the main computer.

### 4.3. Cloud Server

In our work, the cloud server is another important component because it hosts the database module, which stores the post-processing data. The private cloud server plays a critical role in the processes of data storage and public service requests. For the iseAuto shuttle, multiple database architectures were used in the cloud server to store all kinds of data produced by the vehicle. Log data related to the low-level control system, such as braking, steering, and throttle, were stored in a PostgreSQL database. Perceptive data from the sensors were stored in a MySQL database set in parallel in the cloud server. We deploy a similar MySQL database in a remote server to store original sensory and post-processed data collected by prototype, such as camera-frame-projected and radar-enhanced LiDAR data. The database module communicates with the main computer through 4G routers. Moreover, we develop the database in a manner to be able to adapt to other autonomous platforms quickly. There is an interface that allows users to modify the database structure for different sensors and their corresponding configurations. The data that were stored in the database have the labels of the timestamps and path in file systems, which will be useful for the database query tasks. We also deploy this data collection framework onto our autonomous shuttle and publish the data collected by the shuttles when they are on real-traffic duty. The web page interface to access the data is https://www.roboticlab.eu/finest-mobility (accessed on 14 May 2023).

## 5. Performance Evaluation

We developed this dataset collection framework primarily for the purpose of deploying on low-speed urban autonomous vehicles such as autonomous shuttles and food-delivery robots. Perceptive data were collected while autonomous vehicles were performing routine duties. Post-processing such as data decompression, sensor synchronization, and fusion were supposed to be carried out on board. Considering the computational limit of vehicles’ in-built computers, it is critical to evaluate the efficiency of dataset collection framework regarding time and storage space consumption. Please note that the scope of our work is to build a generic practical solution for autonomous vehicles to collect and process perceptive data. The potential usages of the published dataset include scooter speed monitoring, and traffic-sign enhancement, which serve as transportation management for smart cites [72]. Benchmarks for other kinds of autonomous-driving-related research such as object segmentation, tracking, and path completion might benefit from the implementation of this framework, but remain out of the scope of this work.

Table 3 and Table 4 evaluate the performance of this dataset collection framework in our prototype. Table 3 shows the storage occupation and time consumption of the framework’s different modules to process the whole data sequence. The raw data collected from the sensors are stored as ROS bag files. There are two examples listed in this table: the first sequence is the filed-test data collected at the scene where our autonomous shuttles were deployed in Tallinn urban area. The second sequence was recorded at the indoor laboratory. The duration of our tests is 301 and 144 s, corresponding to the size of 3.7 and 0.78 GB. The output of the decompression and fusion operations in our framework are portable network graphics (PNG) images and binary pickle files for each frame, which are explained in detail in Section 3. The final output of our dataset collection framework for these two example sequences is available at https://www.roboticlab.eu/claude/finest_framework/ (accessed on 14 May 2023). As there are two radar sensors installed in our prototype, the ‘radar–LiDAR–camera Fusion’ in Table 3 indicates the time consumption and data size for two radar streams. Please note that the post-processing in our framework was executed in parallel using multiple threads; therefore, the time consumption of the decompression and fusion might vary for different hardware setups and conditions. The data in Table 3 were computed by the main onboard computer of our prototype, which is Intel® NUC 11 with Core™ i7-1165G7 featuring 8 processing threads.

Table 4 shows our evaluation on the framework’s per-frame performance. The first row shows the size of the RGB image and binary LiDAR points per frame. The second row is the sum of the time consumption to produce one image, and one point-cloud binary file since the camera and LiDAR data were synchronized to the same frequency before being forwarded to the post-processing modules. The input of the ’LiDAR Projection’ process is all of the LiDAR point clouds; therefore, this process takes the longest time compared with the other processes.

## 6. Conclusions

In conclusion, this study successfully presents a comprehensive end-to-end generic sensor dataset collection framework for autonomous driving vehicles. The framework includes hardware deploying solutions; sensor fusion algorithms; and a universal toolbox for calibrating and synchronizing camera, LiDAR, and radar sensors. The generality of this framework allows for its application in various robotic or autonomous systems, making it suitable for rapid, large-scale practical deployment. The promising results demonstrate the effectiveness of the proposed framework, which not only addresses the challenges of sensor calibration, synchronization, and fusion, but also paves the way for further advancements in autonomous driving research. Specifically, we showcase a streamlined and robust hardware configuration that maintains ample room for customization while preserving a generic interface for data gathering. Aiming to simplify cross-sensor data processing, we introduce a framework that efficiently handles message synchronization, and low-level data fusion. In addition, we develop a server-side platform allowing for the redundancy of connections from the recording of multiple in-field operational vehicles and the uploading of sensors data. Finally, we feature the framework with the basic web interface allowing one to overview and download the collected data (both raw and processed). Moreover, the framework has the potential for expansion through the incorporation of high-level sensor data fusion, which would enable one to track dynamic objects more effectively. This enhancement can be achieved by integrating LiDAR-camera deep fusion techniques that not only facilitate the fusion of data from these sensors, but also tackle the calibration challenges between LiDAR and camera devices. By integrating these advanced methods, the framework can offer even more comprehensive and efficient solutions for autonomous vehicles, and other applications, requiring the robust and precise tracking of objects in their surroundings. In addition, we view comprehensive evaluations, such as the image quality assessment described by Zhai and Min [73] and the real-traffic object detection benchmark [74] of the results, as future work.

## Figures and Tables

**Table 1:** ROS message types for sensor drivers and calibration processes.

Sensor	Message Type of Topic Published by Driver	Message Type of Topic Subscribed by Calibration Processes
LiDAR Velodyne VLP-32C	velodyne_msgs/VelodyneScan	sensor_msgs/PointCloud2 (LiDAR-camera extrinsic) velodyne_msgs/VelodyneScan (radar-LiDAR extrinsic)
Camera Raspberry Pi V2	sensor_msgs/CompressedImage	sensor_msgs/Image (camera intrinsic) sensor_msgs/Image (LiDAR-camera extrinsic)
Radar TI AWR1843BOOST	sensor_msgs/PointCloud2	sensor_msgs/PointCloud2 (radar intrinsic) sensor_msgs/PointCloud2 (radar-LiDAR extrinsic)

**Table 2:** Specifications of the sensors ion prototype mount.

	FoV (${}^{\circ }$)	Range (m)/Resolution	Update Rate (Hz)
Velodyne VLP-32	40 (vertical)	200	20
Raspberry Pi V2	160 (D)	3280 × 2464	90 in 640 × 480
TI mmwave AWR1843BOOST	100 (H) 40 (V)	4 cm (range resolution) 0.3 m/s (velocity resolution)	10–100

**Table 3:** Data size and time duration of framework’s modules to process the data sequence. The unit of the data size is gigabyte (GB), and the unit of the time is second (s).

	Sequence 1 City Urban	Sequence 2 Indoor Lab
Sequence Duration (s)	301	144
Raw Bag File Size (GB)	3.7	0.78
Synchronization (s)	4.28	1.24
Raw Data Decompressing (s)	0.36	0.09
Raw Data Writing (s)/(GB)	116.63/16.4	54.74/7.4
LiDAR-Camera Fusion (s)/(GB)	510.94/9.2	261.34/4.6
radar–LiDAR–Camera Fusion (s)/(GB)	61.97/5.8	39.38/3.3

**Table 4:** Data size and average time consumption of the framework’s post-processing for each frame. The output of each post-processing is an RGB image with resolution of 1920 × 1080, and the binary pickle file contains the coordinates and the velocity information of the points in each corresponding frame. The unit of the data size is megabyte (MB), and the unit of the time is millisecond (ms).

	Raw Data Decompressing and Writing	LiDAR Projection	Radar-LiDAR Clustering
Size per frame			
RGB image in 1920 × 1080	3 MB	3 MB	3 MB
LiDAR points in binary	1.2 MB	0.9 MB	<0.1 MB
Average time per frame (RGB image in 1920 × 1080 + LiDAR points in binary)	79.7 ms	647.7 ms	108.44 ms

## Data Availability

The source code of the implementation and demonstration of our framework’s prototype is available at https://github.com/Claud1234/distributed_sensor_data_collector (accessed on 14 May 2023). The web interface to access the data collected by the framework that was deployed on real-traffic autonomous shuttle is at https://www.roboticlab.eu/finest-mobility (accessed on 14 May 2023). The final output of our dataset collection framework for two example sequences in Section 5 is available at https://www.roboticlab.eu/claude/finest_framework/ (accessed on 14 May 2023).
